# Application of Transposon Insertion Sequencing to Agricultural Science

**DOI:** 10.3389/fpls.2020.00291

**Published:** 2020-03-18

**Authors:** Belinda K. Fabian, Sasha G. Tetu, Ian T. Paulsen

**Affiliations:** ^1^ARC Centre of Excellence in Synthetic Biology, Macquarie University, Sydney, NSW, Australia; ^2^Department of Molecular Sciences, Macquarie University, Sydney, NSW, Australia

**Keywords:** biocontrol, plant growth promoting bacteria, fertilizer, microbiome, pesticide, transposon insertion sequencing, transposon mutagenesis

## Abstract

Many plant-associated bacteria have the ability to positively affect plant growth and there is growing interest in utilizing such bacteria in agricultural settings to reduce reliance on pesticides and fertilizers. However, our capacity to utilize microbes in this way is currently limited due to patchy understanding of bacterial–plant interactions at a molecular level. Traditional methods of studying molecular interactions have sought to characterize the function of one gene at a time, but the slow pace of this work means the functions of the vast majority of bacterial genes remain unknown or poorly understood. New approaches to improve and speed up investigations into the functions of bacterial genes in agricultural systems will facilitate efforts to optimize microbial communities and develop microbe-based products. Techniques enabling high-throughput gene functional analysis, such as transposon insertion sequencing analyses, have great potential to be widely applied to determine key aspects of plant-bacterial interactions. Transposon insertion sequencing combines saturation transposon mutagenesis and high-throughput sequencing to simultaneously investigate the function of all the non-essential genes in a bacterial genome. This technique can be used for both *in vitro* and *in vivo* studies to identify genes involved in microbe-plant interactions, stress tolerance and pathogen virulence. The information provided by such investigations will rapidly accelerate the rate of bacterial gene functional determination and provide insights into the genes and pathways that underlie biotic interactions, metabolism, and survival of agriculturally relevant bacteria. This knowledge could be used to select the most appropriate plant growth promoting bacteria for a specific set of conditions, formulating crop inoculants, or developing crop protection products. This review provides an overview of transposon insertion sequencing, outlines how this approach has been applied to study plant-associated bacteria, and proposes new applications of these techniques for the benefit of agriculture.

## Introduction

A major component of agricultural ecosystems is the microbiota (bacteria, archaea, protists, fungi, and viruses) present on plants and in soils. Interactions between microbes and plants can be detrimental, commensal, or favorable (Buée et al., 2009). The benefits provided by microbes in agricultural systems include breaking down organic matter, fixing nutrients, making nutrients available for plant use, nutrient recycling, defense against plant pathogens and improving abiotic stress tolerance (Naik et al., 2019). To survive and thrive in their niche, plant-associated microbes need to be able to evade host defenses, use the nutrients available from the host and successfully compete with other microbes (Gonzalez-Mula et al., 2019).

To maximize crop production and minimize losses due to biotic and abiotic stresses it is increasingly clear that we need to take advantage of the benefits that beneficial microbes can provide. However, this is currently difficult to achieve with only a limited understanding of microbial gene functions and their interactions with plants on a molecular level. An increasingly common approach when studying agricultural microbiota is to ascertain microbial community composition using metagenomics (Shelake et al., 2019). This allows for the collection of genetic information about more members of the community than just the microbes that are able to be cultivated (Levy et al., 2018). Decreasing sequencing costs have facilitated the rapid generation of vast amounts of genomic data. However, collecting this sequence information does not readily translate into understanding what functions are being performed within a microbial community and what genes are involved (van Opijnen and Camilli, 2013; Vorholt et al., 2017).

The traditional methods for experimental demonstration of gene function, such as gene knockouts and over-expression, happen at a much slower pace than the rate at which genomic information is accumulating (Chang et al., 2016). The laborious and often problematic nature of this gene function characterization (see section “Saturation Transposon Insertion Mutagenesis and High Throughput DNA Sequencing”) means only a small proportion of genes in a small number of microbes from agricultural systems have been experimentally investigated (Price et al., 2018a). For microbes that have not been experimentally characterized, sequence homology, genome location or similarity of domains is often used to infer gene function, but predictions of gene or organism function generated by these computational approaches are often erroneous, especially when the closest characterized relative is separated by a large phylogenetic distance (Mutalik et al., 2019). There are many circumstances where even these methods geared at prediction of gene function do not yield any functional information and genes remain annotated as hypothetical proteins. The combination of these issues results in the functions of many microbial genes remaining unknown or poorly understood (Van Opijnen and Camilli, 2012; Jarboe, 2018). This is currently limiting our understanding of the role many microbes play in agricultural and other systems and hampers interpretation of microbiome survey data and efforts to formulate optimal plant associated microbial communities (Shelake et al., 2019).

Most of what is currently known about genes involved in beneficial plant interactions has been determined by comparing genomes and individually testing genes that correlate with a phenotype of interest (Levy et al., 2018). A high throughput method of gathering gene functional information and relating genotype to phenotype is required to rapidly advance our understanding of the complex molecular interactions that occur between microbes and plants (van Opijnen and Camilli, 2013; Perry et al., 2016) and move beyond cataloging microbial community members toward a mechanistic understanding of agricultural microbiomes (Poole et al., 2018).

## The Need for Alternatives to Pesticides and Fertilizers

To provide food and fiber for the rapidly growing global population, crop yields need to increase dramatically (Godfray et al., 2012; Pardey et al., 2014). In the past, agricultural intensification has been enabled by the use of pesticides, fertilizers, machinery, and increased plowing depth and sowing density. While this has previously been adequate to increase crop yields and meet global demand (Emmerson et al., 2016), these methods are no longer delivering sufficient increases in yields and are contributing to biodiversity declines which can have flow on effects for agricultural yields, for example, pollination services that are critical for food production (Gallai et al., 2009; Tscharntke et al., 2012). Declines in the rates at which crop yields are increasing, the changing climate and abiotic stresses (especially water availability and salinity) mean that new approaches are necessary to meet demand for food and fiber (Ray et al., 2012; Backer et al., 2018). The major ways to meet this demand while holding agricultural land area stable are to increase crop yields and/or dramatically reduce the quantity of crops lost to disease (Phalan et al., 2011; Godfray et al., 2012).

One path to increasing biomass accumulation and increasing crop yields is through the addition of fertilizers to crop lands. When fertilizers with elevated levels of nitrogen and phosphorus are added to fields they can create an imbalance in the inorganic nutrients present in the soil (Gosal et al., 2018). The addition of phosphorus in this way results in a large proportion binding to soil particles (immobilization) and not being biologically available for plant use (Khan et al., 2007). Excessive use of fertilizers can lead to surrounding lands being contaminated through run-off, changes to the physico-chemical properties of soil and detrimental impacts on the native soil microbiota which can have flow on effects for the resilience of agricultural soils and crop yields (Jangid et al., 2008; Santhanam et al., 2015; Naik et al., 2019). For these reasons, there is increasing recognition that alternatives to inorganic fertilizers are needed (Cordell et al., 2009).

Every year 20–40% of global food production is lost worldwide to plant pests and diseases despite efforts to control crop diseases using pesticides and other crop management techniques (FAO, 2016). Crop diseases are mainly controlled through the use of pesticides and farming practices, such as crop rotation, integrated pest management and the development of disease resistant crop varieties (Syed Ab Rahman et al., 2018). Continuously using pesticides as the major weapon against crop pathogens ultimately results in the pathogens developing resistance and pesticides losing their effectiveness. This has been observed in *Zymoseptoria tritici*, the fungal causative agent for Septoria tritici blotch, progressively evolving resistance to fungicides in Europe since the early 2000s, and since 2011 in Tasmania, Australia (McDonald et al., 2019). The Fungicide Resistance Action Committee (FRAC), a pesticide industry expert group, lists over 429 instances of plant pathogens becoming resistant to fungicides (Fungicide Resistance Action Committee [FRAC], 2018).

In many instances, farmers have to apply higher doses and multiple pesticides to combat this rising resistance; eventually plant pathogens will no longer be controllable using existing pest control methods (Lucas et al., 2015). In addition to rising pathogen resistance, the application of pesticides to crops may cause detrimental off-target effects for many other members of these ecosystems. Commensal and beneficial microbes are often killed and insects that are protecting crops from herbivory or providing essential services such as pollination are damaged or removed from these habitats (Bünemann et al., 2006; Geiger et al., 2010). With the combination of the slowing rate of discovery and development of new pesticides (Borel, 2017), resistance developed by pathogens and the harm pesticides cause to the environment it is widely agreed that new methods of disease control are needed (Syed Ab Rahman et al., 2018).

## Boosting Crop Yields Via Application of Beneficial Bacteria

Many plant-associated bacteria are now understood to have the capacity to positively affect plant growth and there is growing interest in utilizing such bacteria in agricultural settings to reduce reliance on pesticides and fertilizers (Naik et al., 2019). These plant growth promoting bacteria colonize the surface of plants and the thin layer of soil surrounding the plant roots (rhizosphere; Zhang et al., 2017). These bacteria use the nutrients exuded from plant roots to fuel their growth and in turn can stimulate plant growth (Buée et al., 2009).

**FIGURE 1:**
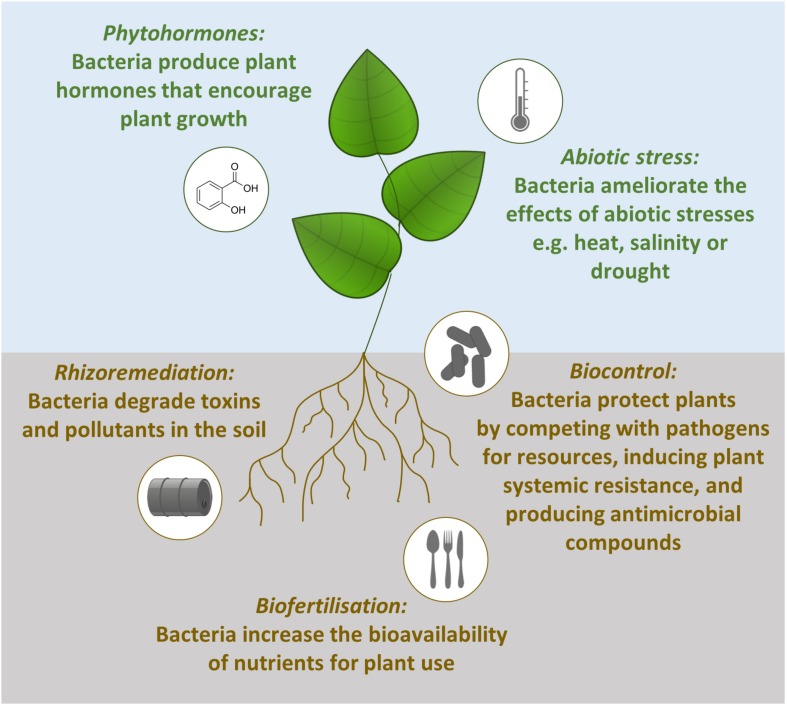
Mechanisms of plant growth promotion by beneficial bacteria. Phytohormone production, abiotic stress tolerance, rhizoremediation and biofertilization are direct methods of plant growth promotion. In contrast, biocontrol is an indirect method of plant growth promotion operating through competition with pathogens, priming plant defenses and producing antimicrobial compounds.

Plant growth is promoted by such bacteria in a number of direct and indirect ways (Figure 1). Firstly, beneficial bacteria can assist with biofertilization, increasing the bioavailability of nutrients for plant use. Examples include atmospheric nitrogen fixation (Ke et al., 2019), solubilizing phosphate (Arkhipova et al., 2019; Wu et al., 2019) and the production of iron binding siderophores which can be taken up by plants (Flores-Félix et al., 2015). The second way rhizobacteria can stimulate plant growth is through the production of phytohormones (also referred to as plant growth regulators). These hormones can stimulate plant growth in the same way as endogenous plant hormones. For example, the endophytic bacteria *Sphingomonas* sp. LK11 releases gibberellins which increases tomato plant shoot length and root weight (Khan et al., 2014). Thirdly, beneficial bacteria can ameliorate the effect of abiotic stresses and lead to increased tolerance of abiotic stresses. The presence of *Pseudomonas putida* AKMP7 has been shown to promote wheat growth under heat stress (Ali et al., 2011) and under waterlogged conditions *Achromobacter xylosoxidans* Fd2 induces waterlogging tolerance which results in 46.5% higher yield from Holy basil plants (*Ocimum sanctum*; Barnawal et al., 2012). The final direct method of promoting plant growth is rhizoremediation. This is when soil bacteria degrade pollutants and attenuate the effects of toxins in the soil. The removal of these contaminants from the environment reduces the stress on the plant and results in increased plant growth (Correa-Garcia et al., 2018). For example, rice growth in heavy metal contaminated soils is boosted by cadmium-resistant *Ochrobactrum* sp. and lead- and arsenic-resistant *Bacillus* sp. (Pandey et al., 2013).

The indirect method for promoting plant growth is when beneficial microbes are used to control plant pathogens, an eco-friendly and sustainable process termed biocontrol (Handelsman and Stabb, 1996). Biocontrol bacteria reduce the impacts that pathogens have on plants through three interacting mechanisms (Figure 1). Firstly, the beneficial microbes grow in the same spaces as the pathogens and compete with them for nutrients and niches. For example, the biocontrol bacteria *Chryseobacterium* sp. WR21 controls bacterial wilt disease by more effectively competing for tomato root exudates compared to the pathogenic bacteria *Ralstonia solanacearum* (Huang et al., 2017). The second method for reducing the impact of plant pathogens is through the induction of plant systemic resistance. *Bacillus amyloliquefaciens* SQR9 primes the immune system of Arabidopsis against the pathogens *Pseudomonas syringae* pv. tomato DC3000 and *Botrytis cinerea* (Wu et al., 2018). The final technique used by plant growth promoting microbes is antibiosis through the production of antibiotics or metabolites. For example, the biocontrol bacteria *Pseudomonas protegens* Pf-5 produces the antibiotic pyoluteorin to protect cotton plants against infection by the oomycete pathogen *Pythium ultimum* (Howell and Stipanovic, 1980).

Consistent and reliable performance in protecting crops against pathogens is an essential characteristic of any plant growth promoting bacteria before consideration for development into a commercial crop protection or crop enhancement product. As a commercial product a bacterial inoculum will be applied in a broad range of settings with diverse physico-chemical parameters. Beneficial bacteria need to not only cope with a range of environments and climatic conditions, but actively enhance plant growth under such conditions. In addition to colonizing the crop host, the bacteria need to compete with existing soil microbiota for resources and be safe for the environment (Rosconi et al., 2016). The bacterial inoculum may be applied in conjunction with chemical pesticides and fertilizers, so it is advantageous if it is able to survive and maintain plant growth promoting activity in the presence of these additions and must also be able to survive packaging, transport and storage before it is applied to a crop (Glick, 2015; Backer et al., 2018). Ideally, the biocontrol agent should also be able to tolerate a range of environmental stresses, for example, drought, salinity, temperature or heavy metal pollution (Compant et al., 2019).

Over 30 bacterial strains have been successfully commercialized into crop inoculants (not including the many *Rhizobia* spp. used for legume crop inoculation; Glick, 2015), but there are many more beneficial strains of bacteria that have potential for development. Even for the beneficial bacteria that have been developed into commercial biocontrol agents, there is often no strong functional understanding of their mechanisms of action (Bardin et al., 2015). There is a clear need for high throughput functional analyses to investigate beneficial bacteria with potential for development into commercial agents (Backer et al., 2018). This would permit much faster identification of the metabolic pathways they use to gather resources, their modes of action in a variety of environments and hosts, and the roadblocks that are limiting their current application.

## Saturation Transposon Insertion Mutagenesis and High Throughput DNA Sequencing

To further our understanding of plant-microbe and microbe-microbe interactions a faster method of determining microbial gene function is needed. The traditional methods of ascribing functions to genes (for example, making single gene knockouts or heterologous expression) are laborious and very slow as mutants are screened individually (Royet et al., 2019). In addition, these methods can be problematic, for example, gene expression in a heterologous bacterial host may exhibit an altered phenotype due to the different genomic context of the original organism (Levy et al., 2018). These methods are also reductionist; mutants are studied in isolation meaning that interactions between genes cannot be identified and genes may not appear important in pure culture even though they may be crucial *in vivo* (Poole et al., 2018).

In the last 10 years, the combination of saturation transposon mutagenesis with high throughput sequencing has enabled identification of the suite of genes and pathways that are important for plant growth promotion in a single experiment (Cole et al., 2017). The fundamental element that underlies this methodology is the transposon; a class of genetic elements that can move to different locations within a genome (Hoffman and Jendrisak, 2002). When a transposon inserts into a genome it may interrupt or modify the function of a gene or regulatory element resulting in a change in the organism’s phenotype, allowing phenotypic changes to be linked to specific gene disruptions (Hoffman et al., 2000; Reznikoff, 2008).

The first step in transposon insertion sequencing is generating a saturated mutant library by using a transposable element to generate a large population of cells with mutations at many locations throughout the genome (Barquist et al., 2013). The choice of transposable element depends on the organism; most commonly used are the Tn*5* or *mariner* transposons (reviewed in Chao et al., 2016). Tn*5* transposons insert randomly into the genome, while *mariner* transposons insert at TA sites. TA site occurrence is relatively regular across the genome but can vary at local scales. Knowing how many possible mariner transposons insertion sites there are in a genome allows for statistical calculations of transposon insertion saturation which is not possible in Tn*5* based mutant libraries. On the other hand, as Tn*5* transposons do not require a specific site for insertion into the genome these libraries can potentially have higher transposon insertion density (Chao et al., 2016).

**FIGURE 2:**
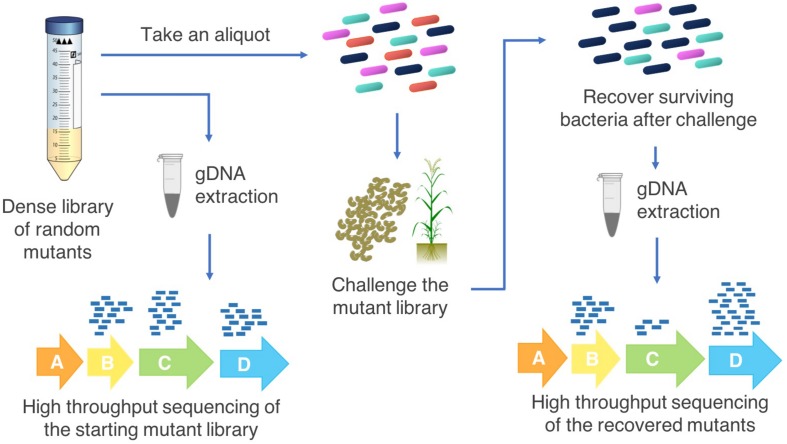
Transposon insertion sequencing methodology for agriculturally relevant bacteria. High throughput sequencing of the starting population of bacterial mutants in the saturated transposon insertion library determines the locations of the transposon insertions in the genome. An aliquot of the mutant library is grown with a selective pressure (for example, persistence on root surfaces in either sterilized or natural soil, pesticide tolerance, abiotic stress), the surviving cells are recovered, and the transposon-chromosome junctions are sequenced. Changes in the abundances of each mutant in the starting pool compared with the challenge pool indicate which genes increase or decrease bacterial fitness under the challenge conditions. This provides information on the possible functions of these genes that can then be investigated by targeted follow up experiments.

A transposon insertion into an essential gene is most often fatal and mutants of these genes will not be included in the resulting mutant library (essentiality is relative to growth conditions; Barquist et al., 2013). Insertions within non-essential genes result in recoverable cells which may show phenotypic or fitness differences under some growth conditions (Hoffman et al., 2000). The goal of this approach is to generate a saturated mutant library of cells which collectively have transposon insertions throughout the genome, enabling assessment of the importance of each gene to fitness under a range of testable growth conditions (Barquist et al., 2016). For example, simultaneous fitness assessment of the hundreds of thousands of mutants in a library is possible by subjecting the mutant library to a defined condition such as exposure to a pesticide or colonization of a host (Chao et al., 2016; Calero et al., 2017; Figure 2).

Multiple methods for transposon insertion sequencing have arisen in the past decade, including TraDIS (Langridge et al., 2009), Tn-Seq (van Opijnen et al., 2009), HITS (Gawronski et al., 2009), and INSeq (Goodman et al., 2009). These methods have been further refined, for example Tn-Seq Circle (Gallagher et al., 2011), RB-TnSeq (Wetmore et al., 2015) and improvements to INSeq (Goodman et al., 2011) to reduce the time and resources required to carry out these studies or improve the effectiveness of the procedure. Other developments, such as TraDISort, enable physical enrichment of desired mutants via cell sorting (Hassan et al., 2016). All of these methods use similar approaches (Figure 2): (1) construct a saturated mutant library by transposon mutagenesis of a bacteria with a transposable element containing an antibiotic resistance cassette; (2) subject the library to challenge assays that affect the fitness of the cells containing mutated conditionally essential genes; (3) DNA extraction from recovered mutants and enrichment for transposon-chromosome junctions; (4) massively parallel sequencing of the mutant library input (pre-challenge) and output (post-challenge) pools; (5) bioinformatic analysis to map the location of each transposon insertion site in the genome; and (6) comparison of the number of reads at each transposon insertion site to determine the conditionally essential genes for the condition of interest.

The differences between the transposon insertion sequencing methods lie in the construction of the mutant library and the enrichment for transposon-chromosome junctions before sequencing. Tn-Seq and INSeq mutant libraries are constructed using mariner transposons. The inclusion of *Mme*I restriction sites at each end of the transposon allows for the chromosomal DNA either side of the transposon to be cut at a fixed length of 20 base pairs during preparation of Tn-Seq and INSeq libraries before sequencing. Adapter ligation follows and PCR and purification steps are carried out. The original INSeq protocol includes a PAGE gel purification step, while Tn-Seq uses agarose gel purification (van Opijnen and Camilli, 2013). Improvements to INSeq purification steps include the use of a biotinylated primer for PCR and magnetic bead purification to decrease the time and cost involved as well as increasing the technique’s sensitivity (Goodman et al., 2011). RB-TnSeq uses random barcodes with the transposon inserted into each cell. This allows for the chromosomal location of the transposon insertion to be mapped from the initial sequencing run and each subsequent sequencing run takes less time and funding (Wetmore et al., 2015). In HITS and TraDIS there is no restriction site included in the transposon, instead random shearing of the genomic DNA is employed, resulting in variable length DNA fragments. In TraDIS, adapters are ligated to all DNA fragments and enrichment of fragments of interest (those containing a transposon-chromosome junction) is achieved through PCR. The HITS technique includes these steps as well as an affinity purification step to purify the PCR amplicons of interest containing the transposon (Gawronski et al., 2009). The details of each method and the advantages and disadvantages have been comprehensively reviewed by Barquist et al. (2013) and van Opijnen and Camilli (2013).

A complementary approach to loss-of-function assays is dual-barcoded shotgun expression library sequencing (Dub-Seq), where gain-of-function overexpression libraries are used in competition assays to gather insights into gene function and the fitness of mutants that have increased gene dosage (Mutalik et al., 2019). Combining data from both gain- and loss-of-function approaches can lead to increased precision of biological insights and help to overcome the limitations when either approach is used in isolation (Mutalik et al., 2019).

The advantage of transposon insertion sequencing approaches is that gene functions can be identified without prior knowledge of their likely roles (Hassan et al., 2016). When using traditional methods of determining gene function, some information about a gene is necessary to establish a starting point for characterization; this can be problematic in situations when sequence databases and genome context do not give many (or any) clues about the function of hypothetical proteins. In contrast, transposon insertion sequencing approaches allow for the simultaneous collection of gene functional information for a large number of genes, even when no other information is available. By comparing the number of transposon-insertion reads in the mutant library before and after a selection pressure is applied the genes involved in coping with that pressure can be identified (diCenzo et al., 2018). Changes in the relative abundances of each mutant shows which genes have been positively or negatively affected by the challenge conditions and therefore their possible functions (Paulsen et al., 2017). Combining information from multiple challenge assays produces multi-dimensional data and leads to a greater functional understanding of both annotated and hypothetical proteins. Such information is useful in selecting candidate genes which are indicated to be required for improved fitness under the conditions of interest for further, targeted functional characterization.

Using a transposon insertion sequencing approach allows for the identification of the genetic factors likely to affect bacterial fitness in a complex condition or environment (Barquist et al., 2013). Transposon insertion sequencing techniques can be used to investigate the suite of genes required for *in vivo* growth in a single experiment, such as assays on plants or plant tissues (Cole et al., 2017). Employing the saturated transposon library in these types of complex, multi-faceted scenarios provides comprehensive information about the genes required to cope with *in vivo* selection pressures, how genes interact and create a complex phenotype, and can reveal the range of modes of action used by bacteria (van Opijnen and Camilli, 2013; Kohl et al., 2019).

To ensure transposon insertion sequencing is used appropriately there are some considerations that need to be taken into account in the design of these high throughput studies. Transposon insertion sequencing allows a mutant library of bacteria to be studied in conjunction with the existing microbiome of an organism to identify genes related to interacting with the host and the host’s microbiome (including beneficial, commensal and pathogenic microbes; Barquist et al., 2013). This technique has been successfully applied in animal models. For example, a *Salmonella enterica* serovar Typhimurium saturated mutant library was screened using chickens, pigs and cattle to identify the genes required for infection in each host (Chaudhuri et al., 2013). When using a dense mutant library in agricultural studies, experiments need to be designed to avoid bottlenecking the population due to insufficient plant surface area or resources to support bacterial growth (van Opijnen and Camilli, 2013). A related consideration when working with bacterial inoculants as part of an existing microbiome is the challenge of recovering enough mutant cells and extracting enough DNA to be able to conduct the high-throughput sequencing. Failure to recover sufficient library after a challenge could lead to biasing of the samples and insufficient sequence depth for reliable results. When analyzing data from transposon insertion sequencing experiments it is important to consider that fitness effects may not be solely due to the gene interrupted by the transposon insertion. If a transposon inserts into an operon or promoter region there could be downstream effects on gene expression.

There are also some limitations to where and how transposon insertion sequencing approaches can be applied. As with other molecular techniques, this approach is only feasible for genetically tractable microbes which can be cultured in the laboratory (Levy et al., 2018). Additional limitations include the inability to identify genes for which there is functional redundancy in the genome (Mutalik et al., 2019) or those encoding “public goods” (products that are secreted and shared with the community; Royet et al., 2019) as these functional deficiencies are unlikely to reduce the fitness of the individuals affected whilst they are in a mixed population (Cole et al., 2017).

The majority of initial studies using transposon insertion sequencing approaches focused on human pathogens, but these methods are starting to be used to examine other microbial lifestyles. Recent studies of human and animal pathogens include susceptibility and resistance of *Escherichia coli* to phage infection (Cowley et al., 2018), the mechanism of antibiotic binding in *Staphylococcus aureus* (Santiago et al., 2018), and resistance of *Borrelia burgdorferi* to reactive oxygen and nitrogen species (Ramsey et al., 2017). Non-pathogenic microbes are also being explored using transposon insertion sequencing methods, for example, regulation and biosynthesis of holdfast assembly has been investigated in *Caulobacter crescentus* (Hershey et al., 2019), circadian clock proteins have been identified in cyanobacteria (Welkie et al., 2018) and previously unknown bacterial amino acid biosynthesis genes have been identified in heterotrophic bacteria (Price et al., 2018b).

## Transposon Insertion Sequencing and the Future of Agriculture

### Current Uses of Transposon Insertion Sequencing for Plant-Associated Microbes

Transposon insertion sequencing techniques have been applied to study a relatively small number of plant-associated bacteria (Table 1). One application has been the discovery of genes critical for leaf, root and rhizosphere colonization (Cole et al., 2017; Liu et al., 2018; Helmann et al., 2019a; Sivakumar et al., 2019). An RB-TnSeq study of *P. syringae* found 31 genes important for colonization of common bean leaf surfaces, including amino acid and polysaccharide biosynthesis genes. Sixty-five genes were important for fitness in apoplastic environments, including those that make up the type III secretion system and syringomycin synthesis genes (Helmann et al., 2019a). Further *in planta* investigation of single gene mutants confirmed that Δ*eftA* and Δ*Psyr_0920* mutants have decreased fitness in the apoplast, suggesting that these glycosyltransferase domain-containing genes are important for the evasion of plant surveillance systems (Helmann et al., 2019a). Investigations of *Pseudomonas simiae* found a large number of amino acid transporter genes are important for survival in the rhizosphere, suggesting that utilizing the amino acids exuded from plant roots confers a selective advantage over microbes that devote resources to the synthesis of these compounds (Cole et al., 2017). Inoculation of wild type and immunocompromised Arabidopsis plants with *Pseudomonas* sp. WC365 identified 231 genes involved in rhizosphere competence (Liu et al., 2018). Follow-up experiments with targeted single gene knockout mutants confirmed the role of the mutants Δ*spuC* and Δ*morA* in inducing pattern-triggered immunity of the Arabidopsis host and inhibiting plant growth. Point mutations in the conserved GGDEF motif of MorA showed that this protein acts as a phosphodiesterase which inhibits biofilm formation (Liu et al., 2018).

**TABLE 1:** Summary of transposon insertion sequencing studies of plant growth promoting and plant pathogenic bacteria.

**Microbe**	**Plant host**	**Method**	**Main finding**	**References**
**Plant and soil colonization**				
*Pseudomonas aeruginosa* PGPR2	Corn	INSeq	Identified 108 genes that contribute to fitness during root colonization-amino acid catabolism, stress adaptation, detoxification, signal transduction, and transport functions	Sivakumar et al., 2019
*Pseudomonas simiae* WCS417r	*Arabidopsis thaliana*	RB-TnSeq	Identified 115 genes required for colonization of the root system and 243 genes where disruption positively affects colonization – large number of these encode proteins for amino acid transport, suggesting that auxotrophy confers a selective advantage in the plant-associated environment rich with exuded amino acids and sugars	Cole et al., 2017
*Pseudomonas syringae* pv. *syringae* B728a	Green bean, *Phaseolus vulgaris*	RB-TnSeq	Genes for amino acid and polysaccharide biosynthesis are important for fitness on the leaf surface and in the leaf interior (apoplast). Genes for type III secretion system and syringomycin synthesis are important in the apoplast	Helmann et al., 2019a
*Bacillus thuringiensis*	Clover, *Triflorium hybridum*	TraDIS	Identified genes required for survival in soil – ability to form spores was a key characteristic for multiplying and colonizing the soil	Bishop et al., 2014
*Pseudomonas sp.* WCS365	*Arabidopsis thaliana*	Tn-Seq	Identified novel factors required to evade plant defenses by conducting screens on wild-type and immunocompromised plants – identified 231 genes that increase rhizosphere fitness and verified seven genes involved in avoiding plant defenses	Liu et al., 2018
**Rhizobial symbiosis**				
*Rhizobium leguminosarum* bv. *viciae* 3841 *Sinorhizobium meliloti* RM1021 *Agrobacterium tumefaciens* UBAPF2	Legumes	INSeq	Used a mariner transposon to mutagenize three members of *Rhizobiaceae* at high frequency. Generated a saturated mutant library in RLV3841 on rich media and identified essential genes	Perry and Yost, 2014
*Rhizobium leguminosarum* bv. *viciae* 3841	Legumes	INSeq	Identified genes required for growth on minimal mannitol containing media. Compared to genes required for growth on rich media and found ∼10% of chromosomal genes required under both growth conditions. Identified plasmid genes encoding functional activities important to central physiology	Perry et al., 2016
*Rhizobium leguminosarum* bv. *viciae* 3841	Legumes	INSeq	Identified genes important for growth on minimal media supplemented with glucose or succinate at both 1 or 21% O_2_. Analyzed central carbon metabolism pathways to examine roles in glucose and succinate metabolism and quantify the ability of pathways to compensate for single mutations	Wheatley et al., 2017
*Sinorhizobium meliloti* RmP3499	Legumes	Tn-seq	Evaluated the interactions between the chromosomal genes and the extra-chromosomal replicons when grown in defined and rich media – ten percent of chromosomal genes found to functionally interact with the replicons	diCenzo et al., 2018
*Sinorhizobium meliloti* 1021	*Medicago truncatula*	Tn-seq	Identified genes and pathways that contribute to sensitivity to NCR247 peptide, a plant signaling peptide with antimicrobial activity which plays a key role during rhizobial differentiation in the nodule – found 78 genes and several pathways that affect sensitivity to NCR247	Arnold et al., 2017
*Sinorhizobium meliloti* Rm2011	Legumes	Signature tagged mutagenesis combined with next generation sequencing	Identified an additional 4,473 transposon insertion sites in a mutant library, bringing the total number of mutants with known insertion sites to 9,562 – 59% of predicted protein-coding genes have a transposon insertion	Serrania et al., 2017
**Pathogenicity and virulence**				
*Dickeya dadantii*	Chicory	Tn-Seq	Identified metabolic pathways and genes required for growth of a necrotrophic pathogen *in planta* – the uridine monophosphate, purine and leucine, cysteine, and lysine biosynthetic pathways are essential for bacterial survival in the plant; RsmC and GcpA are important for regulating the infection process; and glycosylation of flagellin confers fitness during plant infection	Royet et al., 2019
*Salmonella enterica* serovar Typhimurium ATCC 14028	Campari tomato, *Solanum lycopersicum* cv. Campari	Tn-Seq	When colonizing tomatoes, *Salmonella enterica* uses a distinct set of plant-associated genes which only partly overlap with the genes used for virulence in animals and by other phytopathogens during plant infection	de Moraes et al., 2017
*Pantoea stewartii* ssp. *stewartii*	Sweetcorn, *Zea mays* cv. Jubilee	Tn-Seq	*In planta* study showed 486 genes are important for survival (reduced fitness when mutated) and loss of six genes increased fitness. O*mpC, lon* and *ompA* were found to play a role in virulence	Duong et al., 2018
*Agrobacterium tumefaciens*	Tomato, *Solanum lycopersicum*	Tn-Seq	Combined plant metabolomics, transcriptomics and Tn-seq to identify genes and pathways involved in host exploitation. On sucrose the *pgi*, *pycA*, *cisY*, and *sdhCDA* genes are crucial and sucrose breakdown involves the Entner–Doudoroff and tricarboxylic acid (TCA) pathways. When growing on GHB (γ-hydroxybutyric acid) as a carbon source the *blc* genes are crucial and breakdown requires the TCA cycle and gluconeogenesis	Gonzalez-Mula et al., 2019
*Liberibacter crescens* BT-1	Mountain papaya	TraDIS	Identified 238 essential genes in common between *L. crescens* and *L. asiaticus* (unculturable pathogen of citrus) as potential antimicrobial targets	Lai et al., 2016
*Pseudomonas syringae* pv. *actinidiae*	Kiwifruit, *Actinidia* spp.	TraDIS	Identified auxotrophic, motility and lipopolysaccharide (LPS) biosynthesis mutants using a “phenotype of interest” library. Used a “mutant of interest” library to identify a putative LPS mutant	Mesarich et al., 2017
**Bacterial physiology**				
32 bacteria, including eight plant-associated bacteria: *Azospirillum brasilense* Sp245 *Burkholderia phytofirmans* PsJN *Desulfovibrio vulgaris* Miyazaki F *Dyella japonica* UNC79MFTsu3.2 *Herbaspirillum seropedicae* SmR1 *Klebsiella michiganensis* M5a1 *Pseudomonas simiae* WCS417 *Sinorhizobium meliloti* 1021	Wheat roots Onion roots Paddy field Arabidopsis root Cereal root Rice rhizosphere Wheat rhizosphere Alfalfa	RB-TnSeq	Across all 32 bacteria, identified phenotypes for 11,779 genes that are not annotated with a specific function, including 4,135 genes that encode proteins that do not belong to any characterized family in either Pfam or TIGRFAMs. Combined functional associations with comparative sequence analysis to identify putative DNA repair proteins and proposed specific functions for transporter proteins, catabolic enzymes, and domains of unknown function	Price et al., 2018a
*Pseudomonas putida* KT2440		Tn-Seq	Transposon insertion libraries generated in *P. putida* KT2440 and *P. putida* KT2440 Δ*fcs*. *P. putida* found to be significantly more tolerant than *E. coli* to p-coumaric acid, a phenolic acid present in soils and plants. Genes involved in maintenance of membrane structure and efflux of solvent compounds were important when exposed to p-coumaric acid	Calero et al., 2017
*Pseudomonas syringae* pv. *syringae* B728a	Bean, *Phaseolus vulgaris*	RB-TnSeq	Identified and used a hyper-susceptible mutant to identify the substrates of redundant transporters. Identified genes that contribute to tolerance of acridine orange, acriflavine and berberine	Helmann et al., 2019b
*Herbaspirillum seropedicae* SmR1	Rice, maize and sorghum	Tn-Seq	Constructed transposon insertion libraries – identified 395 genes essential for growth in rich media	Rosconi et al., 2016
				
*Pseudomonas putida* KT2440	Soil bacteria	Tn-Seq	Transposon insertion libraries generated in *P. putida* KT2440 and *P. putida* KT2440 Δ*fcs*. *P. putida* found to be significantly more tolerant than *E. coli* to p-coumaric acid, a phenolic acid present in soils and plants. Genes involved in maintenance of membrane structure and efflux of solvent compounds were important when exposed to p-coumaric acid	Calero et al., 2017
*Pseudomonas syringae* pv. *syringae* B728a	Bean, *Phaseolus vulgaris*	RB-TnSeq	Identified and used a hyper-susceptible mutant to identify the substrates of redundant transporters. Identified genes that contribute to tolerance of acridine orange, acriflavine and berberine	Helmann et al., 2019b
*Herbaspirillum seropedicae* SmR1	Rice, maize and sorghum	Tn-Seq	Constructed transposon insertion libraries – identified 395 genes essential for growth in rich media	Rosconi et al., 2016

Another application of transposon insertion sequencing examined interactions between plants and bacteria using legumes and their nitrogen-fixing symbionts, *Rhizobium leguminosarum* and *Sinorhizobium meliloti*. These studies identified genes important for surviving the environmental conditions inside root nodules (Wheatley et al., 2017) and genes that confer resistance to plant antimicrobial peptides (Arnold et al., 2017). Investigations of a previously unknown, but highly conserved, gene *smc03872* showed that it protects *S. meliloti* against the antimicrobial activities of the NCR247 peptide of the host plant *Medicago truncatula*. Site specific mutations in this gene revealed that SMc03872 is a lipoprotein with peptidase activity that can provide its protective effect as long it is anchored in a membrane (Arnold et al., 2017).

Transposon insertion sequencing in pathogenic bacteria can be used to rapidly identify virulence factors, essential genes and modes of action of bacterial pathogens (Table 1). An *in planta* Tn-Seq study of *S. enterica* showed that the colonization of tomatoes uses a distinct set of plant-associated genes which only partly overlap with the genes used for virulence in animals and by other phytopathogens during plant infection (de Moraes et al., 2017). Determining which genes are important for plant host colonization by this bacteria is important as the populations of plant-associated bacteria can act as reservoirs for infection of animal hosts, including humans. Another pathogenic lifestyle is the necrotrophic growth of rot-causing pathogens such as *Dickeya dadantii*. A Tn-Seq study of this pathogen showed that the biosynthetic pathways for uridine monophosphate, purines, and the amino acids leucine, cysteine and lysine, are essential for survival on chicory plants (Royet et al., 2019). The study also determined that the RsmC and GcpA regulators are important in the infection process and glycosylation of flagellin confers fitness during plant infection. Transposon insertion sequencing studies can also identify potential antimicrobial targets, such as genes that are essential for growth of pathogenic bacteria but are not essential in other bacterial strains.

Transposon insertion sequencing studies have also provided insights into the fundamental physiology of plant pathogenic and plant growth promoting bacteria (Table 1). As part of a larger study, Price et al. (2018a) created saturated mutant libraries of eight plant-associated bacteria and conducted fitness testing using multiple carbon and nitrogen sources as well as up to 55 different stress conditions. This study recorded phenotypes for thousands of genes and proposed specific functions for transporter proteins, catabolic enzymes, and domains of unknown function. Transporter substrates (Helmann et al., 2019b) and abiotic and biotic stress tolerance genes (Calero et al., 2017) can also be investigated using these techniques. A Tn-Seq study in *P. putida* KT2440 revealed that genes related to membrane stability are important for p-coumaric acid tolerance (Calero et al., 2017). Follow up studies with single gene knockout mutations showed strong involvement of the cytochrome c maturation system (*ccm* operon) and the *ttg2* operon (encodes an ABC transporter) in this tolerance (Calero et al., 2017). Applying these techniques to a broad range of agriculturally relevant bacteria has the potential to inform efforts to cultivate beneficial plant-microbe interactions and reduce the burden associated with agricultural pathogens.

### Future Avenues to Explore With Transposon Insertion Sequencing

There are a wide variety of ways that the use of transposon insertion sequencing techniques in bacteria could be expanded beyond these current applications to rapidly increase our understanding of bacterial functions relevant for plant growth promotion and agriculture. Bacterial growth in the rhizosphere is influenced by the soil type and structure, soil organic matter, macronutrient levels and moisture levels (Shelake et al., 2019). These physico-chemical properties of the soil can vary at the centimeter scale so a bacterial inoculum applied to a crop may encounter a wide range of these conditions (Fierer, 2017). Bacteria residing in the phyllosphere (above ground plant surfaces) have to be able to cope with widely fluctuating environmental conditions, including light levels, water availability, temperature and UV radiation (Shelake et al., 2019). Transposon insertion sequencing techniques could be applied to plant-associated bacteria to determine which genes are involved in fundamental processes required for living in these environments and what genetic factors make some bacteria highly successful at colonizing these environments.

Bacterial chemotaxis and motility-related genes are essential for detecting signals produced by plants and moving toward chemical attractants or away from deterrents (Scharf et al., 2016). Flagella play a critical role in attachment to plant surfaces and the initial formation of biofilms; both essential processes for biocontrol bacteria to protect plants against pathogens (Nian et al., 2007; Rudrappa et al., 2008). In an agricultural context, bacterial cells with enhanced motility and the ability to form biofilms are better competitors for resources and have greater colonization efficiency, leading to greater enhancement of plant growth (Barahona et al., 2010; Backer et al., 2018; Pinski et al., 2019). Transposon insertion sequencing has successfully been used to identify 14 non-flagellar genes and intergenic regions involved in enhanced motility of *E. coli* EC958, showing that genes outside the flagellar and chemotaxis regulons are important for motility (Kakkanat et al., 2017). This approach could be applied to identify genes important for motility in agriculturally relevant bacteria.

Transposon insertion sequencing can even provide new insights into processes that are extremely well studied. For example, despite decades of sporulation research, a recent Tn-Seq study of *Bacillus subtilis* led to the identification of 24 additional genes involved in sporulation and mutants in which sporulation is delayed or accelerated (Meeske et al., 2016). The genes rhizobial bacteria use to adapt to rhizospheric and root endophytic conditions have been extensively studied, but recent transposon insertion sequencing studies have revealed new insights into the genes important for utilizing carbon compounds exuded by roots and surviving in low oxygen environments inside root nodules (Wheatley et al., 2017).

Most genetic studies of plant-associated bacteria are a snapshot at a particular moment in time and cannot shed light on changing interactions, such as across plant life stages. Executing a succession of transposon insertion sequencing experiments could give insights into the different stages of a plant growth promoting bacteria’s interaction with its host (diCenzo et al., 2019). An *in vivo* transposon insertion sequencing experiment of host infection over a 2-week time series was conducted using *Edwardsiella piscicida*, a fish pathogen. This study examined the fitness of mutants over the course of host infection and identified genes that contribute to colonization of the fish host (Yang et al., 2017). When compared with traditional endpoint transposon insertion sequencing studies the time series experiment identified more genes affecting *in vivo* fitness and yielded new insights into possible targets for vaccine development (Yang et al., 2017). The selective pressures bacteria experience during plant colonization or infection are not constant over time, so using time-series transposon insertion sequencing studies of plant associated bacteria could provide insights into the relative importance of particular genes during these dynamic processes.

When living on plant surfaces plant growth promoting bacteria need to be able to cope with biotic and abiotic stresses, such as plant toxins, anthropogenic pollutants, salinity, acidic or alkaline conditions and temperature. Some of these stresses are commonly experienced in combination, for example, drought and heat stress are often experienced together due to the changing climate. Stress physiologically affects both plants and microbes and can have flow-on effects for plant-microbe interactions (Naik et al., 2019). Transposon insertion sequencing has been successfully used in non-plant-associated organisms to study bacterial responses to stress conditions. A Tn-seq study using high osmolarity, reactive oxygen species and temperature successfully identified individual *E. coli* genes that confer stress resistance as well as gene combinations that work synergistically to impart improved stress resistance (Lennen and Herrgard, 2014). Transposon insertion sequencing analysis of the pathogen *B. burgdorferi* uncovered 66 genes not previously known to be involved in resistance to reactive oxygen and nitrogen species (Ramsey et al., 2017). Conducting *in vitro* or *in planta* transposon insertion sequencing studies that incorporate rhizospheric or phyllospheric stresses could rapidly advance our understanding of the genes employed by plant growth promoting bacteria in stressful conditions.

To extend our knowledge of how genes interact to create a phenotype of interest, transposon mutant libraries could be created using plant-associated bacteria with a genetic background where a specific gene of interest is inactivated. This would allow interactions with the inactivated gene, redundant genes and the components of regulatory networks to be identified (Barquist et al., 2013; van Opijnen and Camilli, 2013). To determine the interaction profiles of five genes of interest in the human pathogen *Streptococcus pneumoniae*, independent transposon mutant libraries were constructed using cells with a background knockout of one of the five genes (van Opijnen et al., 2009). This study revealed interactions that exacerbated or improved fitness defects compared to the single mutants and showed that one of the genes of interest is a master regulator of complex carbohydrate metabolism. Using this technique in plant-associated bacteria could reveal previously unknown gene interactions and shed light on gene networks crucial for growth on plant surfaces.

## Potential Agricultural Applications

Using transposon insertion sequencing allows us to move beyond traditional methods of molecular genetic analyses where bacterial phenotypes and single mutations are linked and perform en masse identification of genes and pathways involved in plant growth promotion (diCenzo et al., 2019). These high-throughput techniques can rapidly accelerate the rate of gene functional determination and provide rapid insights into the genes and pathways that underlie biotic interactions, metabolism and survival of agriculturally relevant bacteria. This will dramatically increase our knowledge of mechanisms that beneficial bacteria use to promote plant growth and the genes and pathways that plant pathogens rely on when causing disease.

Understanding the growth requirements of plant growth promoting bacteria could assist with developing formulations for maximum efficiency when applied to crops (Kohl et al., 2019). For example, if a beneficial bacteria is known to be able to metabolize a particular compound the bacterial inoculant could be formulated to include that compound to create optimal growth conditions for the beneficial bacteria (Syed Ab Rahman et al., 2018; Rocha et al., 2019). This would increase the likelihood of it being able to effectively compete with the native microbiota, colonize the plant surface and persist over time (Naik et al., 2019). A similar effect could be achieved if plant breeding selected crop genotypes that have the ability to support beneficial bacteria, for example by exuding a specific chemical compound from the roots (Syed Ab Rahman et al., 2018; Compant et al., 2019).

By conducting a series of high-throughput transposon insertion sequencing experiments a large amount of information could be leveraged in the design of a bacterial inoculum that is able to promote plant growth across diverse crop species, a broad range of soils, climatic conditions, and abiotic and biotic stresses (Compant et al., 2019). The development of inoculums consisting of multiple bacterial species that use a range of modes of action for biocontrol would reduce the risk of pathogens being able to evolve resistance to a single biocontrol strain (Backer et al., 2018). By incorporating bacteria with a suite of activities a bacterial consortium could be effective across more stages of the plant lifecycle, resulting in increased plant growth. Studies of bacterial consortia have shown that diverse bacterial inoculants show greater survival, reduced levels of plant disease and increased plant biomass (Hu et al., 2016, 2017). Harnessing rapidly generated information about bacterial gene function from transposon insertion sequencing studies has great potential for creating optimized bacterial consortia for crop inoculation across a range of conditions.

Alternative strategies to leverage the plant growth promoting effects of beneficial bacteria are using secreted bacterial metabolites or selecting desirable plant traits. The compounds that beneficial bacteria secrete can act as biostimulants and agricultural soils could be supplemented with these microbial metabolites to trigger plant responses and stimulate plant growth (Backer et al., 2018). The insights from transposon insertion sequencing could uncover ways to boost production of these bioactive secondary metabolites by plant-associated bacteria (Shelake et al., 2019). The findings from transposon insertion sequencing studies could also assist in the identification of desirable plant traits that support the attraction of beneficial bacteria or promote resistance to pathogen colonization. These traits could be selected in plant breeding programs (Syed Ab Rahman et al., 2018; Compant et al., 2019).

If there is a shift in public opinion about genetic engineering, then information gleaned from transposon insertion sequencing studies could be used to direct alterations of plant-associated microbes for the development of crop protection and crop enhancement products. Utilizing information from transposon insertion sequencing to identify targets for gene editing could lead to faster design and development of control methods for crop diseases (Miesel et al., 2003; Royet et al., 2019). Once high throughput analysis rapidly identifies a target gene or pathway, a pathogen could be genetically engineered to create an attenuated or avirulent isolate for use in biological control (Muñoz et al., 2019). These engineered strains can still colonize plants, compete with their pathogenic kin for space and nutrients, and may even induce plant defenses, but they themselves are no longer infectious (Couteaudier, 1992). For example, in glasshouse and field trials *hrpG* mutants of the tomato bacterial spot pathogen *Xanthomonas campestris* pv. *vesicatoria* 75-3 provided up to 76% reduction in disease severity (Moss et al., 2007). Use of genetic engineering in this way could dramatically shorten the timeframe for the development of crop protection products.

Traditional methods of bacterial gene editing and the alteration of protein production, such as gene knockouts and heterologous expression, are now complemented by new gene editing techniques, such as CRISPR-Cas9. Researchers repurposed this bacterial and archaeal cell defense mechanism as a generic gene editing system when they realized that it could be used to recognize and cut specific DNA sequences (Barrangou and Doudna, 2016). As a gene editing mechanism CRISPR-Cas9 is faster and easier to carry out and less expensive than traditional methods, such as homologous recombination (Shelake et al., 2019). Further into the future, synthetic biology and synthetic genomics hold the promise of designing and building bespoke microorganisms capable of specific plant growth promoting functions. Transposon insertion sequencing will play an important role in identifying the genes and pathways that underpin plant growth promotion and will facilitate the design of such synthetic microbes. The performance of these synthetic beneficial microorganisms can be iteratively improved through the classic synthetic biology “design, build, test, learn” cycle (McArthur et al., 2015; Pouvreau et al., 2018).

## Conclusion

We are currently in the midst of a paradigm shift about the role microbes play in our lives. We are transitioning from a simplistic view that microbes are largely disease-causing agents to a more sophisticated understanding of the ubiquitous nature of commensal microbes and the benefits microbes can provide for their hosts. Increasingly society is looking to microbes as a solution for complex problems. Just as research into the human microbiome is revolutionizing medicine and personalized medicine is on the horizon, the future of agriculture may come from rapid advances in our understanding of plant-associated bacterial gene functions and creating tailor made microbial solutions for particular environments, diseases and cropping regimes.

The end goal of these studies is not to have a one-size-fits-all plant growth promoting solution. Conducting transposon insertion studies on plant-associated bacteria will rapidly provide vast amounts of functional information about substantial numbers of agriculturally relevant bacterial genes. As the pressure to use agricultural land most effectively with the least chemical inputs continues to increase, this knowledge will inform agricultural practice so we can increase crop yields and allow us to tackle the challenges of overpopulation and the changing climate.

## Author Contributions

BF wrote the manuscript. ST and IP conceptualized the idea for this work and critically revised the manuscript. BF, ST, and IP approved the final version of the manuscript.

## Conflict of Interest

The authors declare that the research was conducted in the absence of any commercial or financial relationships that could be construed as a potential conflict of interest.

## References

[B1] Ali S. Z. Sandhya V. Grover M. Linga V. R. Bandi V. (2011). Effect of inoculation with a thermotolerant plant growth promoting *Pseudomonas putida* strain AKMP7 on growth of wheat (*Triticum* spp.) under heat stress. *J. Plant Interact.* 6 239–246. 10.1080/17429145.2010.545147

[B2] Arkhipova T. Galimsyanova N. Kuzmina L. Vysotskaya L. Sidorova L. Gabbasova I. (2019). Effect of seed bacterization with plant growth-promoting bacteria on wheat productivity and phosphorus mobility in the rhizosphere. *Plant Soil Environ.* 65 313–319. 10.17221/752/2018-pse

[B3] Arnold M. F. F. Shabab M. Penterman J. Boehme K. L. Griffitts J. S. Walker G. C. (2017). Genome-Wide sensitivity analysis of the microsymbiont *Sinorhizobium meliloti* to symbiotically important, defensin-like host peptides. *mBio* 8:e1060-17. 10.1128/mBio.01060-17. 28765224 PMC5539429

[B4] Backer R. Rokem J. S. Ilangumaran G. Lamont J. Praslickova D. Ricci E. (2018). Plant growth-promoting rhizobacteria: context, mechanisms of action, and roadmap to commercialization of biostimulants for sustainable agriculture. *Front. Plant Sci.* 9:1473. 10.3389/fpls.2018.01473 30405652 PMC6206271

[B5] Barahona E. Navazo A. Yousef-Coronado F. Aguirre de Cárcer D. Martínez-Granero F. Espinosa-Urgel M. (2010). Efficient rhizosphere colonization by *Pseudomonas fluorescens* f113 mutants unable to form biofilms on abiotic surfaces. *Environ. Microbiol.* 12 3185–3195. 10.1111/j.1462-2920.2010.02291.x 20626456

[B6] Bardin M. Ajouz S. Comby M. Lopez-Ferber M. Graillot B. Siegwart M. (2015). Is the efficacy of biological control against plant diseases likely to be more durable than that of chemical pesticides? *Front. Plant Sci.* 6:566. 10.3389/fpls.2015.00566 26284088 PMC4515547

[B7] Barnawal D. Bharti N. Maji D. Chanotiya C. S. Kalra A. (2012). 1-Aminocyclopropane-1-carboxylic acid (ACC) deaminase-containing rhizobacteria protect *Ocimum sanctum* plants during waterlogging stress via reduced ethylene generation. *Plant Physiol. Biochem.* 58 227–235. 10.1016/j.plaphy.2012.07.008 22846334

[B8] Barquist L. Boinett C. J. Cain A. K. (2013). Approaches to querying bacterial genomes with transposon-insertion sequencing. *RNA Biol.* 10 1161–1169. 10.4161/rna.24765 23635712 PMC3849164

[B9] Barquist L. Mayho M. Cummins C. Cain A. K. Boinett C. J. Page A. J. (2016). The TraDIS toolkit: Sequencing and analysis for dense transposon mutant libraries. *Bioinformatics* 32 1109–1111. 10.1093/bioinformatics/btw022 26794317 PMC4896371

[B10] Barrangou R. Doudna J. A. (2016). Applications of CRISPR technologies in research and beyond. *Nat. Biotechnol.* 34 933–941. 10.1038/nbt.3659 27606440

[B11] Bishop A. H. Rachwal P. A. Vaid A. (2014). Identification of genes required by *Bacillus thuringiensis* for survival in soil by transposon-directed insertion site sequencing. *Curr. Microbiol.* 68 477–485. 10.1007/s00284-013-0502-7 24310935

[B12] Borel B. (2017). CRISPR, microbes and more are joining the war against crop killers. *Nature* 543 302–304. 10.1038/543302a 28300126

[B13] Buée M. De Boer W. Martin F. van Overbeek L. Jurkevitch E. (2009). The rhizosphere zoo: an overview of plant-associated communities of microorganisms, including phages, bacteria, archaea, and fungi, and of some of their structuring factors. *Plant Soil* 321 189–212. 10.1007/s11104-009-9991-3

[B14] Bünemann E. K. Schwenke G. D. Van Zwieten L. (2006). Impact of agricultural inputs on soil organisms - a review. *Soil Res.* 44 379–406. 10.1071/SR05125

[B15] Calero P. Jensen S. I. Bojanovič K. Lennen R. Koza A. Nielsen A. T. (2017). Genome-wide identification of tolerance mechanisms towards p-coumaric acid in *Pseudomonas putida*. *Biotechnol. Bioeng.* 15 762–774. 10.1002/bit.26495 29131301 PMC5814926

[B16] Chang Y.-C. Hu Z. Rachlin J. Anton B. P. Kasif S. Roberts R. J. (2016). COMBREX-DB: an experiment centered database of protein function: knowledge, predictions and knowledge gaps. *Nucleic Acids Res.* 44 D330–D335. 10.1093/nar/gkv1324 26635392 PMC4702925

[B17] Chao M. C. Abel S. Davis B. M. Waldor M. K. (2016). The design and analysis of transposon insertion sequencing experiments. *Nat. Rev. Microbiol.* 14 119–128. 10.1038/nrmicro.2015.7 26775926 PMC5099075

[B18] Chaudhuri R. R. Morgan E. Peters S. E. Pleasance S. J. Hudson D. L. Davies H. M. (2013). Comprehensive assignment of roles for *Salmonella* typhimurium genes in intestinal colonization of food-producing animals. *PLoS Genet.* 9:e1003456. 10.1371/journal.pgen.1003456 23637626 PMC3630085

[B19] Cole B. J. Feltcher M. E. Waters R. J. Wetmore K. M. Mucyn T. S. Ryan E. M. (2017). Genome-wide identification of bacterial plant colonization genes. *PLoS Biol.* 15:e2002860. 10.1371/journal.pbio.2002860 28938018 PMC5627942

[B20] Compant S. Samad A. Faist H. Sessitsch A. (2019). A review on the plant microbiome: ecology, functions, and emerging trends in microbial application. *J. Adv. Res.* 19 29–37. 10.1016/j.jare.2019.03.004 31341667 PMC6630030

[B21] Cordell D. Drangert J. O. White S. (2009). The story of phosphorus: global food security and food for thought. *Glob. Environ. Chang.* 19 292–305. 10.1016/j.gloenvcha.2008.10.009

[B22] Correa-Garcia S. Pande P. Seguin A. St-Arnaud M. Yergeau E. (2018). Rhizoremediation of petroleum hydrocarbons: a model system for plant microbiome manipulation. *Microb. Biotechnol.* 11 819–832. 10.1111/1751-7915.13303 30066464 PMC6116750

[B23] Couteaudier Y. (1992). “Competition for carbon in soil and rhizosphere, a mechanism involved in biological control of fusarium wilts,” in *Biological Control of Plant Diseases*, eds Tjamos E. C. Papavizas G. C. Cook R. J. (Boston, MA: Springer), 99–104. 10.1007/978-1-4757-9468-7_13

[B24] Cowley L. A. Low A. S. Pickard D. Boinett C. J. Dallman T. J. Day M. (2018). Transposon insertion sequencing elucidates novel gene involvement in susceptibility and resistance to phages T4 and T7 in *Escherichia coli* O157. *mBio* 9:e00705-18. 10.1128/mBio 30042196 PMC6058288

[B25] de Moraes M. H. Desai P. Porwollik S. Canals R. Perez D. R. Chu W. (2017). *Salmonella* persistence in tomatoes requires a distinct set of metabolic functions identified by transposon insertion sequencing. *Appl. Environ. Microbiol.* 83 1–18. 10.1128/AEM.03028-16 28039131 PMC5311394

[B26] diCenzo G. C. Benedict A. B. Fondi M. Walker G. C. Finan T. M. Mengoni A. (2018). Robustness encoded across essential and accessory replicons of the ecologically versatile bacterium *Sinorhizobium meliloti*. *PLoS Genet.* 14:e1007357. 10.1371/journal.pgen.1007357 29672509 PMC5929573

[B27] diCenzo G. C. Zamani M. Checcucci A. Fondi M. Griffitts J. S. Finan T. M. (2019). Multidisciplinary approaches for studying rhizobium-legume symbioses. *Can. J. Microbiol.* 65 1–33. 10.1139/cjm-2018-0377 30205015

[B28] Duong D. A. Jensen R. V. Stevens A. M. (2018). Discovery of *Pantoea stewartii* ssp. stewartii genes important for survival in corn xylem through a Tn-Seq analysis. *Mol. Plant Pathol.* 19 1929–1941. 10.1111/mpp.12669 29480976 PMC6638119

[B29] Emmerson M. Morales M. B. Oñate J. J. Batáry P. Berendse F. Liira J. (2016). How agricultural intensification affects biodiversity and ecosystem services. *Adv. Ecol. Res.* 55 43–97. 10.1016/bs.aecr.2016.08.005

[B30] FAO (2016). *The State of Food and Agriculture 2016: Climate Change, Agriculture and Food Security.* Rome: FAO.

[B31] Fierer N. (2017). Embracing the unknown: disentangling the complexities of the soil microbiome. *Nat. Rev. Microbiol.* 15 579–590. 10.1038/nrmicro.2017.87 28824177

[B32] Flores-Félix J. D. Silva L. R. Rivera L. P. Marcos-García M. García-Fraile P. Martínez-Molina E. (2015). Plants probiotics as a tool to produce highly functional fruits: the case of *Phyllobacterium* and vitamin C in strawberries. *PLoS One* 10:e0122281. 10.1371/journal.pone.0122281 25874563 PMC4398434

[B33] Fungicide Resistance Action Committee [FRAC] (2018). *List of Plant Pathogenic Organisms Resistant to Disease Control Agents.* Available online at: www.frac.info/docs/default-source/publications/list-of-resistant-plant-pathogens/list-of-resistant-plant-pathogenic-organisms_may-2018.pdf?sfvrsn=a2454b9a_2 (accessed September 21, 2019).

[B34] Gallagher L. A. Shendure J. Manoil C. (2011). Genome-scale identification of resistance functions in *Pseudomonas aeruginosa* Using Tn-seq. *mBio* 2:e00315-10. 10.1128/mBio.00315-10 21253457 PMC3023915

[B35] Gallai N. Salles J. M. Settele J. Vaissière B. E. (2009). Economic valuation of the vulnerability of world agriculture confronted with pollinator decline. *Ecol. Econ.* 68 810–821. 10.1016/j.ecolecon.2008.06.014

[B36] Gawronski J. D. Wong S. M. S. Giannoukos G. Ward D. V. Akerley B. J. (2009). Tracking insertion mutants within libraries by deep sequencing and a genome-wide screen for *Haemophilus* genes required in the lung. *Proc. Natl. Acad. Sci. U.S.A.* 106 16422–16427. 10.1073/pnas.0906627106 19805314 PMC2752563

[B37] Geiger F. Bengtsson J. Berendse F. Weisser W. W. Emmerson M. Morales M. B. (2010). Persistent negative effects of pesticides on biodiversity and biological control potential on European farmland. *Basic Appl. Ecol.* 11 97–105. 10.1016/j.baae.2009.12.001

[B38] Glick B. R. (ed.) (2015). “Introduction to plant growth-promoting bacteria,” in *Beneficial Plant-Bacterial Interactions* (Cham: Springer), 243.

[B39] Godfray H. C. J. Beddington J. R. Crute I. R. Haddad L. Lawrence D. Muir J. F. (2012). The challenge of food security. *Science* 327 812–818. 10.4337/9780857939388 20110467

[B40] Gonzalez-Mula A. Lachat J. Mathias L. Naquin D. Lamouche F. Mergaert P. (2019). The biotroph *Agrobacterium tumefaciens* thrives in tumors by exploiting a wide spectrum of plant host metabolites. *New Phytol.* 222 455–467. 10.1111/nph.15598 30447163

[B41] Goodman A. L. McNulty N. P. Zhao Y. Leip D. Mitra R. D. Lozupone C. A. (2009). Identifying genetic determinants needed to establish a human gut symbiont in its habitat. *Cell Host Microbe* 6 279–289. 10.1016/j.chom.2009.08.003 19748469 PMC2895552

[B42] Goodman A. L. Wu M. Gordon J. I. (2011). Identifying microbial fitness determinants by insertion sequencing using genome-wide transposon mutant libraries. *Nat. Protoc.* 6 1969–1980. 10.1038/nprot.2011.417 22094732 PMC3310428

[B43] Gosal S. K. Gill G. K. Sharma S. Walia S. S. (2018). Soil nutrient status and yield of rice as affected by long-term integrated use of organic and inorganic fertilizers. *J. Plant Nutr.* 41 539–544. 10.1080/01904167.2017.1392570

[B44] Handelsman J. Stabb E. V. (1996). Biocontrol of soilborne plant pathogens. *Plant Cell* 8 1855–1869. 10.1105/tpc.8.10.1855 12239367 PMC161320

[B45] Hassan K. A. Cain A. K. Huang T. Liu Q. Elbourne L. D. H. Boinett C. J. (2016). Fluorescence-based flow sorting in parallel with transposon insertion site sequencing identifies multidrug efflux systems in *Acinetobacter baumannii*. *mBio* 7:e01200-16. 10.1128/mBio.01200-16 27601573 PMC5013296

[B46] Helmann T. C. Deutschbauer A. M. Lindow S. E. (2019a). Genome-wide identification of *Pseudomonas syringae* genes required for fitness during colonization of the leaf surface and apoplast. *Proc. Natl. Acad. Sci. U.S.A.* 116:201908858. 10.1073/pnas.1908858116 31484768 PMC6754560

[B47] Helmann T. C. Ongsarte C. L. Lam J. Deutschbauer A. M. Lindow S. E. (2019b). Genome-wide transposon screen of a *Pseudomonas syringae* mexB mutant reveals the substrates of efflux transporters. *bioRxiv* [Preprint]. 10.1101/684605 31662463 PMC6819667

[B48] Hershey D. M. Fiebig A. Crosson S. Fuqua C. Laub M. T. (2019). A genome-wide analysis of adhesion in *Caulobacter crescentus* identifies new regulatory and biosynthetic components for holdfast assembly. *mBio* 10:e02273-18. 10.1128/mBio.02273-18 30755507 PMC6372794

[B49] Hoffman L. M. Jendrisak J. J. (2002). Transposomes: a system for identifying genes involved in bacterial pathogenesis. *Methods Enzymol.* 358 128–140. 10.1016/S0076-6879(02)58085-X 12474383

[B50] Hoffman L. M. Jendrisak J. J. Meis R. J. Goryshin I. Y. Reznikoff W. S. (2000). Transposome insertional mutagenesis and direct sequencing of microbial genomes. *Genetica* 108 19–24. 10.1023/A:1004083307819 11145416

[B51] Howell C. R. Stipanovic R. D. (1980). Suppression of *Pythium ultimum*-induced damping-off of cotton seedlings by *Pseudomonas fluorescens* and its antibiotic, Pyoluteorin. *Phytopathology* 70 712–715. 10.1094/Phyto-70-712

[B52] Hu J. Wei Z. Friman V.-P. P. Gu S.-H. H. Wang X.-F. F. Eisenhauer N. (2016). Probiotic diversity enhances rhizosphere microbiome function and plant disease suppression. *mBio* 7:e01790-16. 10.1128/mBio.01790-16 27965449 PMC5156302

[B53] Hu J. Wei Z. Weidner S. Friman V. P. Xu Y. C. Shen Q. R. (2017). Probiotic *Pseudomonas* communities enhance plant growth and nutrient assimilation via diversity-mediated ecosystem functioning. *Soil Biol. Biochem.* 113 122–129. 10.1016/j.soilbio.2017.05.029

[B54] Huang J. Wei Z. Hu J. Yang C. Gu Y. Mei X. (2017). *Chryseobacterium nankingense* sp. *nov. WR*21 effectively suppresses *Ralstonia solanacearum* growth via intensive root exudates competition. *BioControl* 62 567–577. 10.1007/s10526-017-9812-1

[B55] Jangid K. Williams M. A. Franzluebbers A. J. Sanderlin J. S. Reeves J. H. Jenkins M. B. (2008). Relative impacts of land-use, management intensity and fertilization upon soil microbial community structure in agricultural systems. *Soil Biol. Biochem.* 40 2843–2853. 10.1016/j.soilbio.2008.07.030

[B56] Jarboe L. R. (2018). Improving the success and impact of the metabolic engineering design, build, test, learn cycle by addressing proteins of unknown function. *Curr. Opin. Biotechnol.* 53 93–98. 10.1016/j.copbio.2017.12.017 29306676

[B57] Kakkanat A. Phan M. D. Lo A. W. Beatson S. A. Schembri M. A. (2017). Novel genes associated with enhanced motility of *Escherichia coli* ST131. *PLoS One* 12:e0176290. 10.1371/journal.pone.0176290 28489862 PMC5425062

[B58] Ke X. Feng S. Wang J. Lu W. Zhang W. Chen M. (2019). Effect of inoculation with nitrogen-fixing bacterium *Pseudomonas stutzeri* A1501 on maize plant growth and the microbiome indigenous to the rhizosphere. *Syst. Appl. Microbiol.* 42 248–260. 10.1016/j.syapm.2018.10.010 30477902

[B59] Khan A. L. Waqas M. Kang S.-M. Al-Harrasi A. Hussain J. Al-Rawahi A. (2014). Bacterial endophyte *Sphingomonas* sp. LK11 produces gibberellins and IAA and promotes tomato plant growth. *J. Microbiol.* 52 689–695. 10.1007/s12275-014-4002-7 24994010

[B60] Khan M. S. Zaidi A. Wani P. A. (2007). Role of phosphate-solubilizing microorganisms in sustainable agriculture — A review. *Agron. Sustain. Dev.* 27 29–43. 10.1051/agro:2006011

[B61] Kohl J. Kolnaar R. Ravensberg W. J. (2019). Mode of action of microbial biological control agents against plant diseases: relevance beyond efficacy. *Front. Plant Sci.* 10:845. 10.3389/fpls.2019.00845 31379891 PMC6658832

[B62] Lai K. K. Davis-Richardson A. G. Dias R. Triplett E. W. (2016). Identification of the genes required for the culture of *Liberibacter crescens*, the closest cultured relative of the *Liberibacter* plant pathogens. *Front. Microbiol.* 7:547. 10.3389/fmicb.2016.00547 27148230 PMC4837290

[B63] Langridge G. C. Phan M. D. Turner D. J. Perkins T. T. Parts L. Haase J. (2009). Simultaneous assay of every *Salmonella Typhi* gene using one million transposon mutants. *Genome Res.* 19 2308–2316. 10.1101/gr.097097.109 19826075 PMC2792183

[B64] Lennen R. M. Herrgard M. J. (2014). Combinatorial strategies for improving multiple-stress resistance in industrially relevant *Escherichia coli* strains. *Appl. Environ. Microbiol.* 80 6223–6242. 10.1128/AEM.01542-14 25085490 PMC4178669

[B65] Levy A. Conway J. M. Dangl J. L. Woyke T. (2018). Elucidating bacterial gene functions in the plant microbiome. *Cell Host Microbe* 24 475–485. 10.1016/j.chom.2018.09.005 30308154

[B66] Liu Z. Beskrovnaya P. Melnyk R. A. Hossain S. S. Khorasani S. O’Sullivan L. R. (2018). A genome-wide screen identifies genes in rhizosphere-associated *pseudomonas* required to evade plant defenses. *mBio* 9:e00433-18. 10.1128/mBio.00433-18 30401768 PMC6222131

[B67] Lucas J. A. Hawkins N. J. Fraaije B. A. (2015). The evolution of fungicide resistance. *Adv. Appl. Microbiol.* 90 29–92. 10.1016/bs.aambs.2014.09.001 25596029

[B68] McArthur G. H. IV Nanjannavar P. P. Miller E. H. Fong S. S. (2015). Integrative metabolic engineering. *AIMS Bioeng.* 2 93–103. 10.3934/bioeng.2015.3.93

[B69] McDonald M. C. Renkin M. Spackman M. Orchard B. Croll D. Solomon P. S. (2019). Rapid parallel evolution of azole fungicide resistance in Australian populations of the wheat pathogen *Zymoseptoria tritici*. *Appl. Environ. Microbiol.* 85 e1908–e1918. 10.1128/AEM.01908-18 30530713 PMC6365823

[B70] Meeske A. J. Rodrigues C. D. A. Brady J. Lim H. C. Bernhardt T. G. Rudner D. Z. (2016). High-throughput genetic screens identify a large and diverse collection of new sporulation genes in *Bacillus subtilis*. *PLoS Biol.* 14:e1002341. 10.1371/journal.pbio.1002341 26735940 PMC4703394

[B71] Mesarich C. H. Rees-George J. Gardner P. P. Ghomi F. A. Gerth M. L. Andersen M. T. (2017). Transposon insertion libraries for the characterization of mutants from the kiwifruit pathogen *Pseudomonas syringae* pv. *actinidiae*. *PLoS One* 12:e0172790. 10.1371/journal.pone.0172790 28249011 PMC5332098

[B72] Miesel L. Greene J. Black T. A. (2003). Genetic strategies for antibacterial drug discovery. *Nat. Rev. Genet.* 4 442–456. 10.1038/nrg1086 12776214

[B73] Moss W. P. Byrne J. M. Campbell H. L. Ji P. Bonas U. Jones J. B. (2007). Biological control of bacterial spot of tomato using *hrp* mutants of *Xanthomonas campestris* pv. *vesicatoria*. *Biol. Control* 41 199–206. 10.1016/j.biocontrol.2007.01.008

[B74] Muñoz I. V. Sarrocco S. Malfatti L. Baroncelli R. Vannacci G. (2019). CRISPR-Cas for fungal genome editing: a new tool for the management of plant diseases. *Front. Plant Sci.* 10:135. 10.3389/fpls.2019.00135 30828340 PMC6384228

[B75] Mutalik V. K. Novichkov P. S. Price M. N. Owens T. K. Callaghan M. Carim S. (2019). Dual-barcoded shotgun expression library sequencing for high-throughput characterization of functional traits in bacteria. *Nat. Commun.* 10:308. 10.1038/s41467-018-08177-8 30659179 PMC6338753

[B76] Naik K. Mishra S. Srichandan H. Singh P. K. Sarangi P. K. (2019). Plant growth promoting microbes: potential link to sustainable agriculture and environment. *Biocatal. Agric. Biotechnol.* 21:101326 10.1016/j.bcab.2019.101326

[B77] Nian H. Zhang J. Song F. Fan L. Huang D. (2007). Isolation of transposon mutants and characterization of genes involved in biofilm formation by *Pseudomonas fluorescens* TC222. *Arch. Microbiol.* 188 205–213. 10.1007/s00203-007-0235-8 17453174

[B78] Pandey S. Ghosh P. K. Ghosh S. De T. K. Maiti T. K. (2013). Role of heavy metal resistant *Ochrobactrum* sp. and *Bacillus* spp. strains in bioremediation of a rice cultivar and their PGPR like activities. *J. Microbiol.* 51 11–17. 10.1007/s12275-013-2330-7 23456706

[B79] Pardey P. G. Beddow J. M. Hurley T. M. Beatty T. K. M. Eidman V. R. (2014). A bounds analysis of world food futures: global agriculture through to 2050. *Aust. J. Agric. Resour. Econ.* 58 571–589. 10.1111/1467-8489.12072

[B80] Paulsen I. T. Cain A. K. Hassan K. A. (2017). Physical enrichment of transposon mutants from saturation mutant libraries using the TraDISort approach. *Mob. Genet. Elem.* 7:e1313805. 10.1080/2159256X.2017.1313805 28580195 PMC5443658

[B81] Perry B. J. Akter M. S. Yost C. K. (2016). The use of transposon insertion sequencing to interrogate the core functional genome of the legume symbiont *Rhizobium leguminosarum*. *Front. Microbiol.* 7:1873. 10.3389/fmicb.2016.01873 27920770 PMC5118466

[B82] Perry B. J. Yost C. K. (2014). Construction of a *mariner*-based transposon vector for use in insertion sequence mutagenesis in selected members of the *Rhizobiaceae*. *BMC Microbiol.* 14:298. 10.1186/s12866-014-0298-z 25433486 PMC4255674

[B83] Phalan B. Balmford A. Green R. E. Scharlemann J. P. W. (2011). Minimising the harm to biodiversity of producing more food globally. *Food Policy* 36 S62–S71. 10.1016/j.foodpol.2010.11.008

[B84] Pinski A. Betekhtin A. Hupert-Kocurek K. Mur L. A. J. Hasterok R. (2019). Defining the genetic basis of plant–endophytic bacteria interactions. *Int. J. Mol. Sci.* 20:E1947. 10.3390/ijms20081947 31010043 PMC6515357

[B85] Poole P. Ramachandran V. Terpolilli J. (2018). Rhizobia: from saprophytes to endosymbionts. *Nat. Rev. Microbiol.* 16 291–303. 10.1038/nrmicro.2017.171 29379215

[B86] Pouvreau B. Vanhercke T. Singh S. (2018). From plant metabolic engineering to plant synthetic biology: the evolution of the design/build/test/learn cycle. *Plant Sci.* 273 3–12. 10.1016/j.plantsci.2018.03.035 29907306

[B87] Price M. N. Wetmore K. M. Waters R. J. Callaghan M. Ray J. Liu H. (2018a). Mutant phenotypes for thousands of bacterial genes of unknown function. *Nat. Chem. Biol.* 557 503–509. 10.1038/s41586-018-0124-0 29769716

[B88] Price M. N. Zane G. M. Kuehl J. V. Melnyk R. A. Wall J. D. Deutschbauer A. M. (2018b). Filling gaps in bacterial amino acid biosynthesis pathways with high-throughput genetics. *PLoS Genet.* 14:e1007147. 10.1371/journal.pgen.1007147 29324779 PMC5764234

[B89] Ramsey M. E. Hyde J. A. Medina-Perez D. N. Lin T. Gao L. Lundt M. E. (2017). A high-throughput genetic screen identifies previously uncharacterized *Borrelia burgdorferi* genes important for resistance against reactive oxygen and nitrogen species. *PLoS Pathog.* 13:e1006225. 10.1371/journal.ppat.1006225 28212410 PMC5333916

[B90] Ray D. K. Ramankutty N. Mueller N. D. West P. C. Foley J. A. (2012). Recent patterns of crop yield growth and stagnation. *Nat. Commun.* 3:1293. 10.1038/ncomms2296 23250423

[B91] Reznikoff W. S. (2008). Transposon Tn*5*. *Annu. Rev. Genet.* 42 269–286. 10.1146/annurev.genet.42.110807.091656 18680433

[B92] Rocha I. D. Ma Y. Souza-Alonso P. Vosatka M. Freitas H. Oliveira R. S. (2019). Seed coating: a tool for delivering beneficial microbes to agricultural crops. *Front. Plant Sci.* 10:1357. 10.3389/fpls.2019.01357 31781135 PMC6852281

[B93] Rosconi F. de Vries S. P. W. Baig A. Fabiano E. Grant A. J. (2016). Essential Genes for *in vitro* Growth of the Endophyte *Herbaspirillum seropedicae* SmR1 as revealed by transposon insertion site sequencing. *Appl. Environ. Microbiol.* 82 6664–6671. 10.1128/aem.02281-16 27590816 PMC5086560

[B94] Royet K. Parisot N. Rodrigue A. Gueguen E. Condemine G. (2019). Identification by Tn-seq of *Dickeya dadantii* genes required for survival in chicory plants. *Mol. Plant Pathol.* 20 287–306. 10.1111/mpp.12754 30267562 PMC6637903

[B95] Rudrappa T. Biedrzycki M. L. Bais H. P. (2008). Causes and consequences of plant-associated biofilms. *FEMS Microbiol. Ecol.* 64 153–166. 10.1111/j.1574-6941.2008.00465.x 18355294

[B96] Santhanam R. Luu V. T. Weinhold A. Goldberg J. Oh Y. Baldwin I. T. (2015). Native root-associated bacteria rescue a plant from a sudden-wilt disease that emerged during continuous cropping. *Proc. Natl. Acad. Sci. U.S.A.* 112 E5013–E5120. 10.1073/pnas.1505765112 26305938 PMC4568709

[B97] Santiago M. Lee W. Fayad A. A. Coe K. A. Rajagopal M. Do T. (2018). Genome-wide mutant profiling predicts the mechanism of a Lipid II binding antibiotic. *Nat. Chem. Biol.* 14 601–608. 10.1038/s41589-018-0041-4 29662210 PMC5964011

[B98] Scharf B. E. Hynes M. F. Alexandre G. M. (2016). Chemotaxis signaling systems in model beneficial plant–bacteria associations. *Plant Mol. Biol.* 90 549–559. 10.1007/s11103-016-0432-4 26797793

[B99] Serrania J. Johner T. Rupp O. Goesmann A. Becker A. (2017). Massive parallel insertion site sequencing of an arrayed *Sinorhizobium meliloti* signature-tagged mini-Tn*5* transposon mutant library. *J. Biotechnol.* 257 9–12. 10.1016/j.jbiotec.2017.02.019 28235609

[B100] Shelake R. M. Pramanik D. Kim J.-Y. (2019). Exploration of plant-microbe interactions for sustainable agriculture in CRISPR Era. *Microorganisms* 7:E269. 10.3390/microorganisms7080269 31426522 PMC6723455

[B101] Sivakumar R. Ranjani J. Vishnu U. S. Jayashree S. Lozano G. L. Miles J. (2019). Evaluation of InSeq to identify genes essential for *Pseudomonas aeruginosa* PGPR2 corn root colonization. *G3* 9 651–661. 10.1534/g3.118.200928 30705119 PMC6404608

[B102] Syed Ab Rahman S. F. Singh E. Pieterse C. M. J. Schenk P. M. (2018). Emerging microbial biocontrol strategies for plant pathogens. *Plant Sci.* 267 102–111. 10.1016/j.plantsci.2017.11.012 29362088

[B103] Tscharntke T. Clough Y. Wanger T. C. Jackson L. Motzke I. Perfecto I. (2012). Global food security, biodiversity conservation and the future of agricultural intensification. *Biol. Conserv.* 151 53–59. 10.1016/j.biocon.2012.01.068

[B104] van Opijnen T. Bodi K. L. Camilli A. (2009). Tn-seq: high-throughput parallel sequencing for fitness and genetic interaction studies in microorganisms. *Nat. Methods* 6 767–772. 10.1038/nmeth.1377 19767758 PMC2957483

[B105] Van Opijnen T. Camilli A. (2012). A fine scale phenotype-genotype virulence map of a bacterial pathogen. *Genome Res.* 22 2541–2551. 10.1101/gr.137430.112 22826510 PMC3514683

[B106] van Opijnen T. Camilli A. (2013). Transposon insertion sequencing: a new tool for systems-level analysis of microorganisms. *Nat. Rev. Microbiol.* 11 435–442. 10.1038/nrmicro3033 23712350 PMC3842022

[B107] Vorholt J. A. Vogel C. Carlström C. I. Müller D. B. (2017). Establishing causality: opportunities of synthetic communities for plant microbiome research. *Cell Host Microbe* 22 142–155. 10.1016/j.chom.2017.07.004 28799900

[B108] Welkie D. G. Rubin B. E. Chang Y. G. Diamond S. Rifkin S. A. LiWang A. (2018). Genome-wide fitness assessment during diurnal growth reveals an expanded role of the cyanobacterial circadian clock protein KaiA. *Proc. Natl. Acad. Sci. U.S.A.* 115 E7174–E7183. 10.1073/pnas.1802940115 29991601 PMC6064986

[B109] Wetmore K. M. Price M. N. Waters R. J. Lamson J. S. He J. Hoover C. A. (2015). Rapid Quantification of Mutant Fitness in Diverse Bacteria by Sequencing Randomly Bar-Coded Transposons. *mBio* 6:e00306-15. 10.1128/mBio.00306-15 25968644 PMC4436071

[B110] Wheatley R. M. Ramachandran V. K. Geddes B. A. Perry B. J. Yost C. K. Poole P. S. (2017). Role of O2 in the Growth of *Rhizobium leguminosarum* bv. *viciae* 3841 on Glucose and Succinate. *J. Bacteriol.* 199:e572-16. 10.1128/JB.00572-16 27795326 PMC5165102

[B111] Wu F. Li J. Chen Y. Zhang L. Zhang Y. Wang S. (2019). Effects of phosphate solubilizing bacteria on the growth, photosynthesis, and nutrient uptake of *Camellia oleifera* abel. *Forests* 10:348 10.3390/f10040348

[B112] Wu G. Liu Y. Xu Y. Zhang G. Shen Q. Zhang R. (2018). Exploring elicitors of the beneficial Rhizobacterium *Bacillus amyloliquefaciens* SQR9 to induce plant systemic resistance and their interactions with plant signaling pathways. *Mol. Plant Microbe Interact.* 31 560–567. 10.1094/MPMI-11-17-0273-R 29309236

[B113] Yang G. Billings G. Hubbard T. P. Park J. S. (2017). Time-resolved transposon insertion sequencing reveals genome-wide fitness dynamics during infection. *mBio* 8:e01581-17. 10.1128/mBio.01581-17 28974620 PMC5626973

[B114] Zhang R. Vivanco J. M. Shen Q. (2017). The unseen rhizosphere root–soil–microbe interactions for crop production. *Curr. Opin. Microbiol.* 37 8–14. 10.1016/j.mib.2017.03.008 28433932

